# Prevalence of Overweight and Obesity in France: The 2020 Obepi-Roche Study by the “Ligue Contre l’Obésité”

**DOI:** 10.3390/jcm12030925

**Published:** 2023-01-25

**Authors:** Annick Fontbonne, Andrew Currie, Patrick Tounian, Marie-Christine Picot, Olivier Foulatier, Marius Nedelcu, David Nocca

**Affiliations:** 1 Centre of Clinical Investigation, Inserm, Montpellier University Hospital, 34295 Montpellier, France; 2 Centre for Epidemiology and Population Health, Inserm, Paris-Saclay University, USQV, 94805 Villejuif, France; 3 Department of Upper GI Surgery, Epsom & St Helier NHS Trust, Epsom KT18 7EG, UK; 4 Saint Eloi Hospital, Montpellier University Hospital, 34295 Montpellier, France; 5 Pediatric Nutrition and Gastroenterology Department, Trousseau Hospital, APHP, Sorbonne University, 75571 Paris, France; 6 Clinical Research and Epidemiology Unit (Department of Medical Information), Inserm, Montpellier University Hospital, 34295 Montpellier, France; 7 Scientific Council, “Ligue Contre l’Obésité” (League Against Obesity), 75008 Paris, France; 8 ELSAN, Saint Michel Clinic, Surgical Centre for Obesity, 83100 Toulon, France; 9 ELSAN, Bouchard Clinic, 13006 Marseille, France; 10 Institute for Functional Genomics, UMR 5023 CNRS-U1191, Inserm, Montpellier University, 34295 Montpellier, France

**Keywords:** overweight, obesity, prevalence, France

## Abstract

Obepi-Roche 2020 by the “Ligue Contre l’Obésité” (League Against Obesity) estimated overweight and obesity prevalence in France. The adopted methodology was chosen to be as similar as possible to that of a series of quota-based surveys conducted every three years from 1997 to 2012 (Obepi-Roche studies). The 2020 survey was conducted online from 24th September to 5th October 2020 by the Odoxa polling institute on a sample of metropolitan French subjects aged 18 years or over. Participants (*n* = 9598) self-measured their height and weight according to detailed instructions. Prevalence estimates were produced for all categories of body mass index. The prevalence of excess weight was 47.3% (17.0% of subjects with obesity), with higher values in the north and east of France. When comparing these 2020 estimates to previous Obepi-Roche estimates in order to visualize trends since 1997, it appeared that overweight fluctuated around 30%, and obesity prevalence increased steadily at a rapid pace. The increase was even steeper in the youngest age groups and for severe and complex obesity. Given the slightly different methodologies between the 1997–2012 studies and the 2020 survey, the worrying trends in obesity prevalence since 1997 must be confirmed, calling for a reedition of the Obepi-Roche series.

## 1. Introduction

Overweight and obesity are some of the greatest health and economic risks worldwide [1,2]. In addition to the noncommunicable diseases they have long been known to cause, from cardiometabolic complications to cancers [3], the COVID-19 pandemic revealed yet another threat, as people suffering from excess weight were also more likely to experience severe outcomes of the infection [4,5]. The recent WHO report on obesity in Europe [3] indicated that 23% of the European region are living with obesity. Additionally, it underlined that despite several initiatives to curb the obesity epidemic since the launch of the Agenda for Sustainable Development in 2015 [6], there has been no progress in halting the rise in prevalence, as well as in the prevalence of associated conditions, such as type 2 diabetes.

In France, the prevalence of overweight and obesity was estimated every three years from 1997 to 2012 by a recurring survey based on a representative sample of the adult population (18 years or over), named the Obepi-Roche studies. These were conducted by the French polling institute Kantar-Sofres under the supervision of Inserm and Assistance Publique-Hôpitaux de Paris and financed by Roche pharmaceutical company; the financing stopped in 2012, ending the series of studies. The first surveys evidenced a sharp increase in obesity prevalence, but the last edition indicated a possible flattening of the curve, with a prevalence of 15.0% in 2012 compared to 14.5% in 2009 [7,8].

Since then, only a few other estimates on national samples have been produced. The Constances study, a large ongoing cohort of French Social Security insurance recipients aged 18–69 years, found a prevalence of obesity around 14% in 2013, which increased slightly to a little more than 15% in 2016 [9]. In another large-scale study on a randomly selected sample of the French population, the Esteban study, the prevalence in 2015, for adults aged 18–74 years, was 17.2% [10]. These differing estimates, although not discordant, are consequences of the varying study designs, subject selection and anthropometric measures in the three surveys, which prevent straightforward comparisons.

Considering the importance of monitoring overweight and obesity trends, particularly in view of the differing WHO and French estimates, the non-governmental organisation “Ligue Contre l’Obésité” (League Against Obesity) decided to launch the present survey on the model of the Obepi-Roche 1997–2012 studies. The aim was to provide an update to their results in order to produce comparable estimates for France over the long term.

## 2. Materials and Methods

### 2.1. Study Design and Participants

The Obepi-Roche 2020 survey by the “Ligue Contre l’Obésité” (LCO 2020 Obepi-Roche) was conducted by the polling institute Odoxa. From a large panel of volunteers registered online for the purpose of answering polls, the institute contacted a sample of metropolitan French subjects aged 18 years or over, built by the quota-sampling method, i.e., based on the distribution of the national references for age, sex, occupation and educational level, after a double stratification by region and city size.

Panelists meeting the inclusion criteria (i.e., metropolitan French subjects aged 18 years or over) were e-mailed an invitation to participate requiring them to self-measure their weight, height and waist circumference as detailed in illustrated instructions attached to the e-mail. A following e-mail was sent 24 h later with a link directing to a short online questionnaire (see Supplementary File). Returns of questionnaires were closely monitored by the polling institute. Panelists not responding within 48 h were excluded from participation, and an invitation e-mail was sent to another panelist in the same quota category. Subjects returning questionnaires received a gratification in the form of gift coupons.

The online survey took place between 24 September and 5 October 2020. Polling was stopped at about 10,000 returned questionnaires. Questionnaires returned by women who declared they were pregnant or had given birth in the past three months were discarded, as were questionnaires with missing values for weight and/or height.

### 2.2. Variables of Interest

Weight and height as measured by the surveyed subjects were used to calculate their body mass index (BMI), which was then categorized according to the WHO definition [11]: underweight (BMI < 18 kg/m^2^), normal weight (18 kg/m^2^ ≤ BMI < 25 kg/m^2^), overweight (25 kg/m^2^ ≤ BMI < 30 kg/m^2^), class I/moderate obesity (30 kg/m^2^ ≤ BMI < 35 kg/m^2^), class II/severe obesity (35 kg/m^2^ ≤ BMI < 40 kg/m^2^), class III/severe and complex obesity (BMI ≥ 40 kg/m^2^). This article will define the combined overweight and obesity population as people with excess weight.

In addition to variables recorded for the quota sampling (e.g., sex, age, occupation, region), participants were asked if they were currently being or had been treated in the past for the following conditions: hypertension, diabetes, high blood cholesterol, cardiovascular diseases such as stroke or myocardial infarction, cancer, obstructive sleep apnoea syndrome, gastroesophageal reflux disease, osteoarthritis, depression or other psychological problems.

### 2.3. Descriptive Strategy

The final sample reflected, by design, the characteristics of the French metropolitan population aged 18 and over, with only slight deviations that generated weighting coefficients applied to crude means or percentages to correct for representativity.

These means and percentages served to draw the distribution of subjects between BMI categories, to show the prevalence of excess weight and obesity by sex, age group, professional category and region, and to compute the prevalence of associated past or current morbidities by BMI category. The LCO 2020 Obepi-Roche study remained purely descriptive, and no statistical test was performed.

Subsequently, the results of the LCO 2020 Obepi-Roche study were added graphically to the results produced by the six previous Obepi studies, conducted every three years from 1997 to 2012 with the same aim of estimating prevalence of overweight and obesity in France [7,8,12]. They were based, like the LCO 2020 Obepi-Roche study, on quota-built representative samples of French households in which all members aged 18 years or over (except pregnant women) were asked to participate. For each of these previous studies, questionnaires were sent by posted mail to 20,000 households from the polling institute panel, and return rates with complete measurements of height and weight oscillated between 59 and 70%, depending on the year [8]. The obtained graphs of the trends from 1997–2012 with added 2020 estimates were again merely descriptive and interpreted visually without statistical procedures.

## 3. Results

### 3.1. Sample Characteristics of the LCO 2020 Obepi-Roche Study

The polling institute received 9598 valid answers to the online questionnaire for adults. As for the general French population that the sample represented by its quota design, mean age was around 50 years (49.1 ± 16.7 years) with a weighted male/female ratio of 91.2% (crude ratio: 86.6%). Pensioners, students, unemployed or inactive people represented 41.5% (crude ratio: 43.4%), and people with a current occupation were more or less equally divided between the categories of factory workers (12.7%), clerks (16.9%), intermediate professions (14.7%) and managers or equivalent (14.2%).

Mean height was 170.0 cm (176.6 cm for men and 163.9 cm for women), an increase of +1.3 cm since Obepi 2012, and mean weight was 74.1 kg (81.2 kg for men and 67.3 kg for women), an increase of +1.6 kg since Obepi 2012. Mean BMI was 25.5 kg/m^2^ (26.0 kg/m^2^ for men and 25.1 kg/m^2^ for women), which constitutes a small increase of +0.1 kg/m^2^ since Obepi 2012.

### 3.2. Prevalence of Overweight and Obesity in the LCO 2020 Obepi-Roche Study

**Figure 1:**
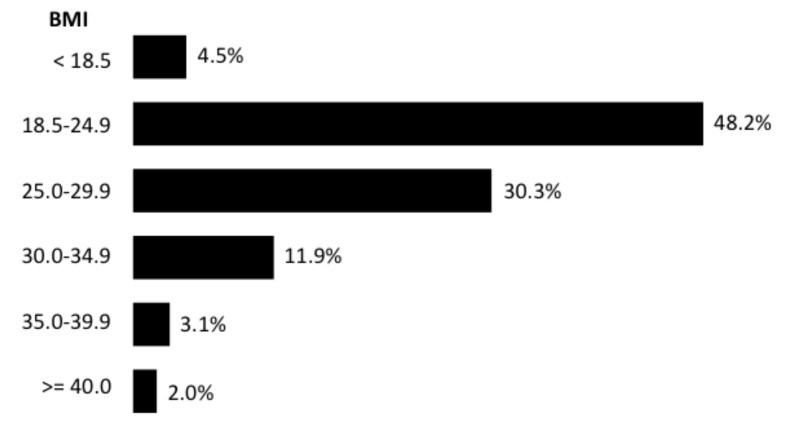
Distribution of subjects by BMI category, LCO 2020 Obepi-Roche study.

The distribution along BMI categories is shown in Figure 1. Altogether, 17.0% of subjects were obese, and the prevalence of people with excess weight was 47.3%.

Prevalence of excess weight was higher in men (53.5%) than in women (41.3%), yet it was the contrary for the prevalence of obesity: 16.7% for men and 17.4% for women.

Prevalence of excess weight was also higher with increasing age: 23.2% for subjects aged 18 to 24 years, 35.2% (25–34 years), 44.0% (35–44 years), 50.7% (45–54 years), 57.2% (55–64 years) and 57.3% (65 years and over). Prevalence of obesity in 2020 in the same age groups was respectively: 9.2%, 13.8%, 16.7%, 18.4%, 19.2% and 19.9%.

Prevalence of excess weight was also dependent on socioprofessional factors, with a negative relation to the qualification of the occupation: it was 51.1% for factory workers or equivalent, 45.3% for clerks, 43.0% for people in intermediate jobs and 35.0% for managers or people in equivalent positions. Prevalence of obesity in 2020 in the same categories was respectively: 9.9%, 14.4%, 17.8% and 18.0%.

### 3.3. Regional Distribution of Obesity in the LCO 2020 Obepi-Roche Study

**Figure 2:**
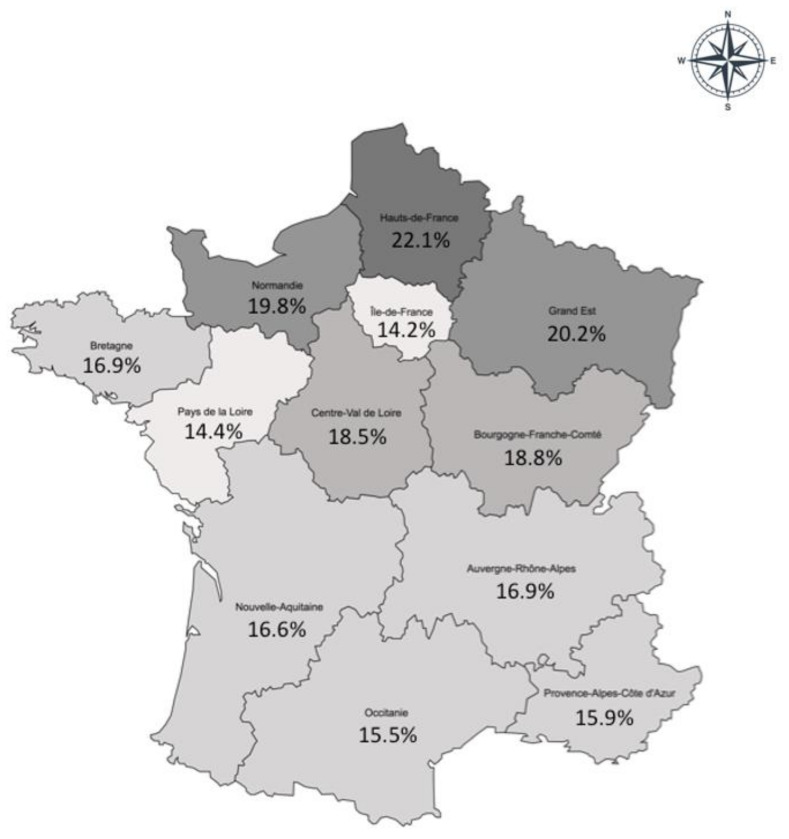
Regional distribution of the prevalence of obesity (BMI ≥ 30 kg/m^2^), LCO 2020 Obepi-Roche study.

The prevalence of obesity was highest (more than 20%) in the north and northeastern regions of France, and it was lowest (less than 14.5%) in the region around Paris (Ile-de-France) and the lower Loire (Pays de la Loire) region (Figure 2). Excepting this latter region and its neighbour, Britanny (Bretagne), there was a north-south decreasing gradient in obesity prevalence.

### 3.4. Associated Comorbidities

The prevalence of hypertension, diabetes and sleep apnoea syndrome increased greatly and linearly in a function of overweight and increasing degrees of obesity (Table 1). For high blood cholesterol, cardiovascular diseases, osteoarthritis and depression or other psychological disorders, prevalence started to rise at the stage of obesity (BMI ≥ 30 kg/m^2^), and slopes were not as marked. For these pathologies, it was even observed that prevalence was close or even superior in persons with a BMI lower than 25 kg/m^2^ compared to overweight ones. A higher prevalence of gastroesophageal reflux disease was also observed in normal weight compared to overweight subjects, and even allowing for a slight increasing trend of prevalence along increasing degrees of obesity, prevalence in subjects with class III/severe and complex obesity was lower than prevalence in normal weight individuals. Prevalence of past or current cancer was highest in subjects with BMI lower than 25 kg/m^2^ and did not evidence a trend across the other classes of BMI.

### 3.5. Evolution of the Estimates between Obepi-Roche 1997–2012 and the LCO 2020 Obepi-Roche Study

**Figure 3:**
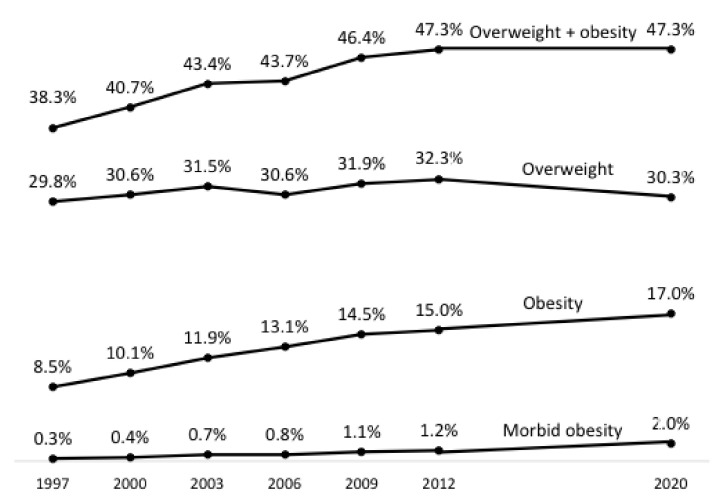
Position of LCO 2020 Obepi-Roche estimates of overweight and/or obesity compared to the Obepi studies 1997–2012 [8]. Overweight: 25 kg/m^2^ ≤ BMI < 30 kg/m^2^; obesity: BMI ≥ 30 kg/m^2^; severe and complex (“morbid”) obesity: BMI ≥ 40 kg/m^2^.

The prevalence of excess weight in the Obepi studies of 1997–2012 increased at a rapid pace between 1997 and 2009 but less so between 2009 and 2012, suggesting the possibility of a plateau [8]. The Obepi 2020 results confirmed this plateau, since the prevalence in 2020 was exactly the same as in 2012 (Figure 3). However, the stable figure is due to a decrease in the prevalence of overweight, whereas the prevalence of obesity has increased substantially, continuing the trend already evidenced in the 1997–2012 Obepi studies. The trend for overweight, on the contrary, when considering all the estimates since 1997, appeared rather to be fluctuating around 30%.

**Figure 4:**
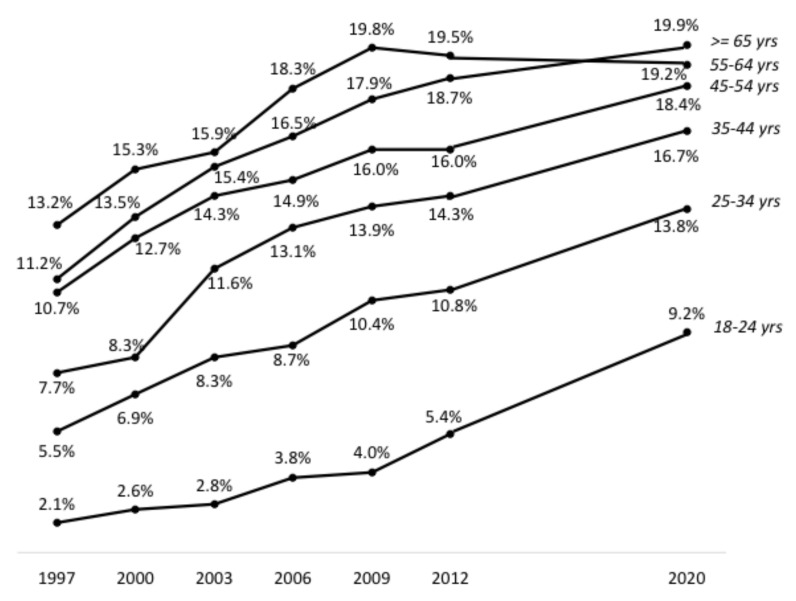
Evolution of the prevalence of obesity (BMI ≥ 30 kg/m^2^) by age groups, Obepi-Roche 1997–2012 [8] with addition of LCO 2020 Obepi-Roche study.

When compared to previous Obepi studies, trends in the prevalence of obesity by age groups were all increasing, except for the 55–64 years category, for which the trend has been decreasing since Obepi 2009 (Figure 4). By contrast, the upward trend was especially marked for the youngest age group (18–24 years).

**Figure 5:**
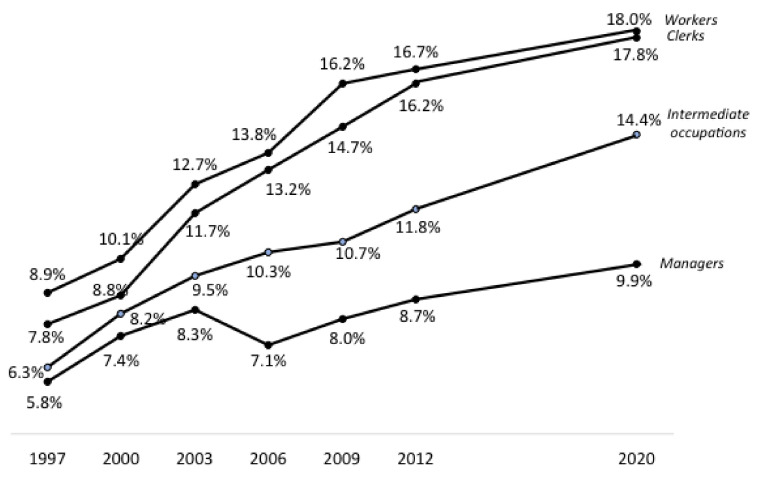
Evolution of the prevalence of obesity (BMI ≥ 30 kg/m^2^) by professional category, Obepi-Roche 1997–2012 [8] with addition of LCO 2020 Obepi-Roche study.

The trends in the prevalence of obesity over all Obepi studies were also clearly influenced by work qualification (Figure 5): close and rapidly increasing for workers and clerks, with a narrowing of their difference over time, increasing less steeply for people in intermediate occupations, and more or less stable since Obepi 2000 for managers, except for a small increase between 2012 and 2020.

As for regional distribution, all previous Obepi studies have shown a higher prevalence in the north and northeast of France and generally a lower prevalence in Britanny and lower Loire, but the decreasing north-south gradient was not always as visible as in the LCO 2020 Obepi-Roche study [8].

## 4. Discussion

The 2020 edition of the Obepi-Roche studies by the “Ligue Contre l’Obésité” showed that almost half the French population were people with excess weight, with 17% being obese (BMI ≥ 30 kg/m^2^) and 2% suffering from severe and complex obesity (BMI ≥ 40 kg/m^2^). The observed prevalence of obesity was close to those found by the Constances cohort in 2016 (15%) [9] and the Esteban study in 2015 (17.2%) [10].

Compared to the European Region [3], these results situate France in a less alarming situation than the average, since the WHO overall estimates are substantially higher: 59% for excess weight and 23% for obesity. In line with the Obepi 2020 results, overweight in Europe was found to be more prevalent in men than in women, whereas it was the opposite for obesity. Further, obesity prevalence was higher in people with lower educational attainment, a well-known finding consistent with Obepi 2020 observations by professional category.

The prevalence of obesity worldwide has been estimated by the GBD 2015 Obesity Collaborators to be around 12% among adults [13]. Again, the authors found that “the prevalence of obesity was generally higher among women than among men in all age brackets”. Of course, the variability was extreme, with estimates higher than 30% for countries such as the USA or Saudi Arabia, whereas in India and most sub-Saharan African countries, obesity prevalence was much lower than 10%. Globally, Europe had a higher prevalence in northern than in southern countries, with France having estimates close to those of Italy or Spain.

As for trends, the LCO 2020 Obepi-Roche study confirmed the plateauing of the prevalence of excess weight in France, which had already been observed in the last two Obepi surveys, in 2009 and 2012 [8]. However, when overweight and obesity were considered separately, the curve for overweight seemed to fluctuate around 30% over the whole period, from 1997 to 2020, whereas the prevalence of obesity increased steadily, doubling from 8.5% in 1997 to 17.0% in 2020. As for severe and complex obesity prevalence, it was multiplied by almost seven over the same time period.

These are worrying trends, especially when considering that the increase in obesity prevalence was even steeper in the younger age groups. This is in line with the 2013 to 2016 findings of the Constances study, which evidenced a significant increase in obesity only in young adults [9]. In the same study, contrary to the LCO 2020 Obepi-Roche study, the increase in obesity between 2013 and 2016 was restricted to class I obesity, whereas severe and severe and complex obesity prevalence exhibited nonsignificant decreasing trends. It is worth noting that the Constances cohort, aged 18–69 years, is on average younger than the Obepi sample, and that its representativeness of the French population depends on complex weighting and calibration procedures, to correct for biases due to differential non-participation [9]. On the other hand, participants’ weight and height were measured by trained personnel in Social Security centres and not self-measured as in Obepi. It is known that, at least in high-income countries, self-reported height is generally higher, and self-reported weight lower, than objective performed measures [14,15]. This results in an underestimation of BMI, which in the case of the Obepi studies, would mean that overall and severe and complex obesity prevalence is even higher compared to the Constances estimates. However, as previously stated, the designs, subject selection and data collection of the Obepi and Constances studies are so different that it is hazardous to make comparisons [16].

Obesity prevalence in the Obepi studies, which almost doubled over 20 years, is obviously rising at a higher rate than the 21% increase that the WHO European Region report estimated for the 10 years before 2016 [3]. The GBD 2015 Obesity Collaborators found that obesity prevalence has continuously increased in most countries since 1980, and that the rates of increase for adults were higher for the youngest age groups [13]. These observations support the conclusions of the WHO report on obesity in Europe [3], that progress has still to be made for halting the rise in obesity prevalence, and they call for the reinforcement of policies and actions to prevent obesity, with a focus on younger people.

### Limitations and Strengths

The Obepi-Roche studies’ main limitations are the voluntary participation of subjects issued from polling panels, aiming at representing the French population but possibly differing on unmeasured characteristics by the simple fact that they agreed to enter the panel and be contacted for surveys; and the already mentioned self-measurement of height and weight, which made Charles, principal investigator of the 1997 to 2012 Obepi studies, advise “considering the estimates produced by the Obepi surveys as minimum estimates for obesity in France” [17].

They also have several strengths. Although the quota method is considered less rigorous than random selection of the population to guarantee representativeness, it minimizes non-responses that often create biases in more traditionally obtained samples and/or impose correction procedures of uncertain validity. Further, studies based on the quota method are cheaper and relatively simple to carry out, which makes them more easily repeatable, an asset for monitoring rapidly changing trends and the reason why this method was used in the 1997–2012 Obepi studies. Of note, the LCO 2020 Obepi-Roche study also relied on the same quota selection method and strived to keep its methodology as close as possible to the first series of studies, in order to produce results that could validly continue previously observed trends. However, there were unavoidable differences in the Internet era: the panels from which the participants were drawn are on-line communities, replacement of non-respondents is easier and quicker, and of course, invitations to participate were sent by e-mail, and questionnaires were accessed and completed through a website. It is not known to which degree these technical differences impact the final population of participants in the current study, but when compared to the French population, their characteristics were very close to full representativeness, as in the earlier Obepi studies.

## 5. Conclusions

The 1997–2012 Obepi-Roche surveys were important epidemiological studies that provided reliable representative indicators of obesity prevalence in France. Though other large-scale epidemiological investigations have been completed in France since 2012, there has been a paucity of studies offering similar methodology to the Obepi surveys and therefore providing some longitudinal perspective. The launching of a new edition, hopefully the first of a new series, by the “Ligue Contre l’Obésité”, has importantly complemented other large-scale studies that have been developed in France since the 2012 Obepi survey with a different methodology [9,10,16,18,19].

The main findings were that although the prevalence of excess weight (overweight and obesity) appeared to be plateauing, the prevalence of obesity was actually increasing and at a much more rapid rate than that which the WHO estimated for the European region [3]. Moreover, the slope was steeper in younger generations and for more severe degrees of obesity, calling for the reinforcement of policies and actions to prevent obesity, with a focus on younger people. It would be important to confirm these observations in another Obepi-Roche survey using exactly the same design as the 2020 edition, considering that the comparison to the 1997 to 2012 series is not entirely rigorous given the slight methodological differences. This is projected to be done in 2023.

## Figures and Tables

**Table 1:** Prevalence of specific past or current morbidities by BMI category, LCO 2020 Obepi-Roche study.

	<25 kg/m^2^	≥25 and <30 kg/m^2^	≥30 and <35 kg/m^2^	≥35 and <40 kg/m^2^	≥40 kg/m^2^
Hypertension	17.3%	25.1%	34.5%	39.3%	43.2%
Diabetes	6.0%	9.8%	18.4%	21.3%	31.1%
High blood cholesterol	18.8%	18.6%	21.6%	21.0%	31.9%
Cardiovascular disease	7.0%	6.9%	7.6%	9.1%	11.6%
Cancer	12.0%	6.8%	7.2%	5.3%	8.0%
Sleep apnoea syndrome	7.3%	7.7%	15.1%	22.5%	30.9%
Gastroesophageal reflux disease	24.2%	18.2%	21.4%	22.6%	22.2%
Osteoarthritis	18.5%	14.7%	17.5%	22.7%	22.6%
Depression, psychological disorders	25.0%	11.9%	16.6%	18.2%	24.9%

## Data Availability

The dataset analyzed during the current study is available from the corresponding author on reasonable request.

## Supplementary Materials

The following supporting information can be downloaded at: https://www.mdpi.com/article/10.3390/jcm12030925/s1, Supplementary File: Questionnaire adressed to 18 years and +.
