# Supplementary material for: Prevalence of Overweight and Obesity in France: The 2020 Obepi-Roche Study by the “Ligue Contre l’Obésité”
Source: J Clin Med. 2023 Jan 25;12(3):925. doi: 10.3390/jcm12030925 (PMC9918095; doi:10.3390/jcm12030925)
Supplement: Supplementary file 1 [file jcm-12-00925-s001.zip › jcm-2106801-supplementary.pdf]

Supplementary File

Questionnaire Obepi

Adults

Questionnaire adressed to 18 years and +

I. Questionnaire TNS 2012 adapted

- Explanatory note on the measures

Could you indicate…

Q1. Your current height (measured today)
Numerical response in cm

Q2. Your current weight (measured today)
Numerical response in kg

Q3. Your waist circumference (measured today)
Numerical response in cm

Body shape

Q4. Which body shape do you think is closest to yours?

Q5. What is your ideal body shape?

(TO WOMEN)

Q6. Are you currently pregnant or have you given birth in the last 3 months?

- Yes
- No

Q7. Currently, do you smoke?

- Yes
- No, you stopped smoking
- No, you never smoked

[To those who have quit smoking]

Q8. Has quitting smoking caused weight gain?

- Yes
- No

Q9. Are you currently being treated or have you been treated for…
(several possible answers)

High blood pressure (or hypertension)
Diabetes
Cancer
Sleep apnea syndrome

Osteoarthritis
Depression or psychological disorders
High cholesterol
Cardiovascular pathology (stroke or infarction)
Gastroesophageal reflux disease (GERD) or heartburn

- You are currently being treated
- You have been treated
- No

Q10. Have you had surgery to treat a weight problem with gastric band, bypass (short circuit) or sleeve? (Several possible answers - except "no" -)

- Gastric band
- Bypass
- Sleeve
- Other
- No

[If a surgery in Q10]
Q11. When did you have this operation?
(specify the month (optional) and the year)
N.B. : If you have had more than one, please specify the month and year of the last surgery

Numerical answer type XX/XXXX

Q12. In the last 12 months, have you consulted a physician for a weight problem?
- Yes
- No

Q13. In your household, would you rather say that, financially speaking? (one possible answer)
- You are comfortable/it's okay
- It's tight
- You can hardly manage
- You can't manage without going into debt

II. Questions COVID 19

COVID19

Q14. Regarding the Covid-19 virus, have you been confronted with any of the following situations: (several possible answers)
- You think you have had COVID-19
- You have been diagnosed positive with COVID-19
- You have been hospitalized for COVID-19
- You went to intensive care for COVID-19

- Nothing of the sort

III. Questions about discrimination

Q15. Have you ever experienced or are you experiencing discrimination?

1. Yes, you have experienced discrimination
2. Yes, you are currently experiencing discrimination
3. No

[If "yes currently", i.e. item 2 in Q15]

Q16. How often do you experience discrimination?

- Everyday
- At least twice a week
- At least once a week
- At least once per month
- Some times per year
- Less often

[If 2 in Q15]

Q17. And what types of discrimination do you encounter? Discrimination...
(Several possible answers)

- In the medical field
- In the educational and/or professional field
- Inside the family
- In the public space

IV. Questions « Regions »

Q18. In your opinion, are the following infrastructures sufficiently accessible to people with large sizes?

Public transportation
Colleges
Universities
Sports and physical activity equipments
Cultural places
Bars et restaurants

- Yes
- No

Q19. Do you think that local products are a guarantee of better food?

- Yes, certainly
- Yes, probably
- No, probably not
- No, certainly not

Q20. Of the themes below, what do you think should be the main concern for our country in the coming months?

- Employment
- Purchasing power
- Education
- Environment
- Health
- Security
